# The Link Between Periodontal Disease and Rheumatoid Arthritis: An Updated Review

**DOI:** 10.1007/s11926-014-0408-9

**Published:** 2014-01-24

**Authors:** Joanna Koziel, Piotr Mydel, Jan Potempa

**Affiliations:** 1 Department of Microbiology, Faculty of Biochemistry, Biophysics, and Biotechnology, Jagiellonian University, ul. Gronostajowa 7, 30-387 Krakow, Poland; 2 Broegelmann Research Laboratory, The Gade Institute, University of Bergen, Bergen, Norway; 3 Oral Health and Systemic Diseases Research Group, University of Louisville School of Dentistry, Louisville, KY USA

**Keywords:** Periodontal disease, Rheumatoid arthritis, Citrullination, PPAD, Periodontitis, *P. gingivalis* peptidyl-arginine deiminase (PPAD)

## Abstract

*Porphyromonas gingivalis* is a leading pathogen in chronic periodontitis, a disease process involving progressive destruction of the tissues that support the teeth. Recently, the organism has been reported to produce a unique bacterial enzyme, *P. gingivalis* peptidyl-arginine deiminase (PPAD), which has the ability to convert arginine residues in proteins to citrulline. Protein citrullination alters protein structure and function; hence, PPAD may be involved in deregulation of the host’s signalling network and immune evasion. Further, accumulating evidence suggests a role for autoimmunity against citrullinated proteins in the development of rheumatoid arthritis (RA). As inflammatory conditions in the lungs of cigarette smokers contribute to the breakdown of immune tolerance to citrullinated epitopes, chronic exposure to citrullinated proteins at periodontitis sites may also predispose susceptible individuals to the development of autoantibodies and the initiation of RA. In this review, we discuss evidence that PPAD may represent a mechanistic link between periodontitis and RA, diseases that are known to be significantly associated at the epidemiological level.

## Introduction

Rheumatoid arthritis (RA) and periodontal disease (PD) are two common chronic inflammatory diseases affecting humans worldwide. In 2003, the total cost attributable to arthritis and other rheumatic conditions in the United States was $128 billion, equivalent to 1.2 % of the 2003 U.S. gross domestic product (http://www.cdc.gov/arthritis/data_statistics). Furthermore, 23 % of the US population age 65+ has been reported to suffer from severe PD. Together, development of both diseases brings considerable consequences for public health and for the quality of life of affected individuals.

There may be a non-causal association between PD and RA due to shared genetic and environmental risk factors, such as expression of the MHC class II *HLA-DRB1* allele and smoking, respectively [1–5]. Despite differences in initiating etiological mechanisms, evidence emerging from numerous clinical and epidemiological studies suggests an association between RA and PD [6–9]. Compared to the general population, subjects with PD are at an increased risk of developing RA, and vice versa; PD is at least 2-fold more prevalent in patients with RA. In addition, the clinical course of PD in RA patients is more severe and is independent of age, gender, ethnicity, or smoking history, as compared to non-RA individuals. Furthermore, RA and PD utilise similar effector destructive mechanisms, in that the inflammatory cells and proinflammatory cytokines that drive chronic bone erosion in RA and chronic gum destruction in PD are similar. Current novel and exciting findings strongly support the idea that PD could be a factor in the initiation and maintenance of the autoimmune inflammatory responses that occur in RA [10].

## *Porphyromonas gingivalis* is a Key Pathogen in Periodontitis

Periodontitis, the chronic inflammation of the supporting tissues surrounding the teeth, is one of the most prevalent inflammatory diseases of mankind. Tooth loss is common in severe forms of the disease and has been reported to afflict more than 20 % of the human population [11]. Further, a paradigm is emerging linking periodontitis with the development of atherosclerosis [12] and RA [7, 9, 13, 14].

It is now generally accepted that chronic periodontitis is initiated by the colonisation of dental plaque by a set of pathogenic bacteria, especially *P. gingivalis*, *Tannerella forsythia*, and *Treponema denticola*. These periodontal pathogens, referred to as the “red complex” due to their strong association with the disease [15], are well equipped with a broad array of virulence factors. *P. gingivalis* expresses lipopolysaccharide (LPS), fimbrae, and haemagglutins, which enable the bacterium to colonise and invade periodontal pockets, and is therefore known as the “master manipulator” of the host homeostatic system [16]. Its most potent weapons are, however, extracellular cysteine proteases, referred to as gingipains, which allow the bacterium to use the host innate immune response to its own benefit [17, 18]. Because its gingipains render it resistant to complement, *P. gingivalis* directly benefits from activation of the complement pathway, which initiates and maintains the inflammatory reaction [19]. In addition, degradation of antimicrobial peptides by gingipains allows other pathogenic bacteria co-aggregating with *P. gingivalis* to persist in the gingiva. Moreover, gingipains affect proinflammatory signalling pathways by cleavage and activation of the proteinase-activated receptor-2 (PAR-2) on human neutrophils. Futile attempts by the host immune response to eliminate infection subsequently lead to connective tissue damage, including alveolar bone resorption [20, 21].

## Role of Citrullination in RA Development

It is now clear that the majority of RA cases are triggered by an autoimmune response to citrullinated proteins. Such proteins are generated under physiological conditions, but the loss of tolerance in genetically susceptible individuals initiates the generation of autoantibodies against citrullinated proteins (ACPA) in the synovia and the subsequent development of RA [22–24].

Protein citrullination by enzymatic deimination of the guanidine group of an arginine side chain is the post-translational modification that converts positively charged peptidyl-arginine to neutral peptidyl citrulline. Protein citrullination is essential for many physiological processes, including terminal differentiation of the epidermis (pro-filaggrin and keratin), brain development [myelin basic protein (MBP)], and regulation of gene expression via chromatin remodeling [25, 26]. Apart from being involved in many physiological processes, citrullination also occurs under the pathological inflammatory conditions of excessive cellular apoptosis, necrosis, and netosis. In the latter process, hypercitrullination of histones is needed for formation of neutrophil extracellular traps, which are part of the innate immune system response to bacterial infection [26]. Thus, deimination has been linked to multiple sclerosis, psoriasis, Alzheimer’s disease, primary open-angle glaucoma, obstructive nephropathy, and RA.

The citrullination process leads to alterations in intra- and inter-molecular interactions of protein targets possessing Arg residues essential for their structure. This may alter the three-dimensional architecture of modified proteins and their solubility in water, and may lead to the generation of neo-epitopes, thus breaching immunological tolerance to citrullinated proteins. In susceptible individuals, the process may initiate a cascade of events leading to the induction of RA [22, 23, 27]. Antibodies to ACPA are known to be a sensitive and specific marker that can be detected years before the clinical onset of the disease. During the clinical course of RA, their presence and serum levels strongly correlate with disease severity. A recent report reveals elevated levels of ACPA in patients with aggressive periodontitis [28]. Furthermore, RA patients diagnosed as ACPA-positive were more likely to have moderate to severe periodontitis than ACPA-negative RA patients [29]. Because the ACPA response is peptidyl citrulline-specific, host enzymes which catalyse the protein modifications that lead to the generation of citrullinated epitopes are considered highly important targets for drug development.

## PPAD is a Unique Bacterial Enzyme among Peptidyl-arginine Deiminases

Protein citrullination is carried out by peptidyl-arginine deiminases (PADs; enzyme commission number (EC) 3.5.3.15). The activity of mammalian PAD enzymes is dependent on high concentrations of calcium (Ca^2+^); thus, citrullination is likely to occur in conditions that lead to the mobilisation of free intracellular calcium, such as chemokine receptor ligation, cell death, and differentiation. Five different PADs (PAD1, 2, 3, 4, and 6) identified in humans are encoded by five paralogous genes clustered on chromosome 1p35–36. PAD1 and PAD3 are mainly found in epidermis and hair follicles; PAD2 is expressed in a variety of tissues, including muscle, brain, and haematopoietic cells; and human PAD4 (formerly known as PAD5) is found primarily in haematopoietic cells. PAD homologues for some or all of these enzymes have also been found in other mammals, with similar genomic organisation across species [30].

*P. gingivalis* is unique among periodontal pathogens in its expression of peptidyl-arginine deiminase (PPAD). Some enzymatic properties of PPAD differ significantly from those of the human PADs. Specifically, PPAD is active at a higher pH and does not require calcium for activity. PPAD also citrullinates C-terminal arginine residues and deiminates free arginine, while mammalian PADs lack these activities [31]. The process of modification of C-terminal arginines is facilitated by colocalisation of PPAD in the outer membrane with arginine-specific gingipains (RGPs), cysteine proteases of *P. gingivalis* [32••]. Gingipains cleave protein chains, exposing C-terminal arginines that are rapidly citrullinated by PPAD, which generates polypeptides with citrullinated Arg located at the C-terminus. Arg-gingipain-null mutants of *P. gingivalis* are devoid of citrullination, confirming that the citrullination of surface proteins is dependent on the activity of arginine gingipain proteases. Furthermore, recent data revealed that PPAD citrullinates peptides generated by the degradation of fibrinogen and α-enolase by *P. gingivalis* gingipains [33]. Citrullination of fibrin and vimentin by PPAD was also described. Citrullination of bradykinin, another PPAD substrate, at the C-terminus [31] may interfere with kinin proinflammatory activity. Finally, PPAD deiminates the C-terminal Arg in epidermal growth factor, abrogating this cytokine’s biological activity [34]. PPAD is clearly expressed in vivo and is immunogenic in mice, as we were able to detect antibodies against PPAD in animals inoculated with a wild-type strain of *P. gingivalis* (W83) but not with the PPAD-null isogenic mutant [35••].

Recent findings indicate that infection with *P. gingivalis* precedes RA and that the bacterium is a likely factor in the initiation and maintenance of the autoimmune inflammatory responses that occur in this disease [10]. In this respect, the presence of *P. gingivalis*-PAD (PPAD), an enzyme expressed by *P. gingivalis* but absent in other prokaryotes [36], may have a profound impact on the development and progression of RA via citrullination of proteins to generate neo-epitopes.

## Citrullination by PPAD as a Putative Mechanistic Link between PD and RA

*P. gingivalis* is the only known microorganism to produce PAD, and in inflamed *P. gingivalis*-infested periodontitis sites, PPAD may set in motion a chain of events that breaks immunotolerance to citrullinated proteins and leads to the development of RA. In line with this hypothesis, ACPA titres in RA patients have been shown to correlate with the presence of PD [29]. Moreover, Lappin et al. recently revealed that ACPA titres are higher in patients with PD compared to healthy donors [37•].

### Host Epitopes Modified by PPAD

Aberrant citrullination has been observed in RA; however, it is still unclear whether citrullination in RA creates novel epitopes or uncovers cryptic ones in susceptible individuals. Among the potential autoantigens in RA that are efficiently citrullinated by human PADs are fibrinogen, enolase, vimentin, and collagen II (CII) [38, 39, 40•]. PPAD localised on the bacterial surface is perfectly positioned to citrullinate host proteins interacting with *P. gingivalis* via bacterial adhesins. In keeping with this hypothesis, *P. gingivalis* rapidly generates fibrinogen-derived peptides with carboxy-terminal citrulline residues [33]. A B cell-dominant epitope of α-enolase termed citrullinated enolase peptide-1 (CEP-1) was also identified as an autoantigen in RA. Recent data revealed that enolase is a substrate for PPAD. Furthermore, CEP-1 shows 82 % sequence similarity with *P. gingivalis* α-enolase [41]. Therefore, CEP-1 antibodies cross-react with the equivalent epitope of *P. gingivalis*-derived enolase. An increased level of antibodies specific for CEP-1 was detected in the chamber fluid of mice infected with *P. gingivalis* [35••]. Finally, pathological changes typical of arthritis were induced in DR4-IE transgenic mice immunised with citrullinated human and *P. gingivalis* α-enolase [40•].

### PPAD Autocitrullination

*P. gingivalis* efficiently citrullinates its own proteins, and subcellular fractionation revealed that the majority of such proteins are associated with the periplasm, and with the outer and inner membrane fractions [33]. Although PPAD preferentially citrullinates C-terminal arginine residues, recent data revealed a striking feature of PPAD: it undergoes autocitrullination [31, 42]. Mass spectrometry analysis revealed citrullination of 7 out of 18 arginines in the PPAD polypeptide chain, all of which were internal [32••]. Therefore, PPAD as a citrullinated bacterial protein itself constitutes a potent antigen that may break the tolerance to citrullinated host proteins. This hypothesis was recently supported by the identification of IgG specifically recognising citrullinated PPAD in sera from RA patients. A *P. gingivalis* strain that expresses catalytically inactive PPAD and is thus devoid of autocitrullination (PPAD^C351A^) did not induce an antibody response, confirming a specific immune response to citrullinated PPAD [32••]. Moreover, the significantly elevated antibody response to PPAD, but not RgpB, in sera from RA patients compared to sera from control and PD patients indicates that PPAD could be an antigen relevant to the pathogenesis of RA. The specificity of the immune response to citrullinated PPAD was confirmed by the reactivity of RA sera to multiple synthetic citrullinated peptides spanning the PPAD polypeptide chain. This study revealed the presence of antibodies to 10 of the 13 tested citrullinated peptides, specifically in RA sera [32••]. Two PPAD peptides (CPP3 and CPP8) were reactive in 40 % of RA samples, indicating their possible application for screening for anti-PPAD antibodies in future epidemiological studies. Taken together, the above observations may suggest that *P. gingivalis* infection can uniquely prime the immune response in RA due to the appearance of autocitrullinated PPAD. The potential pathophysiologic role of autocitrullinated PPAD in RA opens up a novel area for future investigations.

### In Vivo Role of PPAD

In vivo studies have revealed a relationship between joint disease and PD, as mice with pre-existing periodontitis developed more severe arthritis at a faster rate than control animals [43, 44•]. Moreover, there is an increasing body of evidence showing that the severity of periodontitis is related to the progression of RA, and that the profile of oral microbiota in patients with new-onset RA is extremely similar to that of patients suffering from chronic RA [45, 46]. Lastly, using a collagen-induced arthritis model (CIA), it was demonstrated for the first time that infection with *P. gingivalis* not only exacerbates CIA but also appears to play a role in sensitising animals to early disease development [35••]. The arthritis that occurred in mice infected with *P. gingivalis* was characterised by significantly greater bone and cartilage destruction in the affected joints. These clinical signs of arthritis manifested significantly earlier and were accompanied by a more severe disease course than in non-infected animals. The early onset and aggravated progression of CIA occurred only in animals inoculated with viable *P. gingivalis*. Infection with *P. intermedia*, another important pathogen associated with PDs [15], had no effect on the course of CIA. Similar to infection with *P. intermedia*, inoculation with either heat-killed *P. gingivalis* or the purified cell membrane fraction from the same organism had no effect on either the rate or the severity of CIA. Taken together, these findings indicate the requirement for live *P. gingivalis,* which releases bacterial factors exerting a direct or indirect (via stimulation of host systems) effect on the host that ultimately triggers an autoimmune reaction. This hypothesis also drew on recent findings in which immunity to *P. gingivalis*, but not to *P. intermedia* (or *Fusobacterium nucleatum*), was shown to be significantly associated with the presence of RA-related autoantibodies in individuals at risk for the disease [47•].

Recent data revealed that chronic *P. gingivalis* oral infection prior to arthritis induction increases Th17 cell immune responses, including significant secretion of cytokines such as IL-1β, IL-6, IL-22, tumor necrosis factor-α, transforming growth factor-β, and IL-23. This observation suggests that chronic oral infection may influence RA development mainly through the activation of Th17-related pathways [48].

The induction of ACPA is dependent on deiminase activity; thus, PAD expressed by *P. gingivalis* is a potential risk factor in RA development. Recent data revealed that inoculation of animals with an isogenic *P. gingivalis*-PAD knockout strain (ΔPPAD) prior to immunisation with CII had no influence on clinical CIA development or progression [35••]. Histologically, ΔPPAD inoculation had no effect on the degree of synovitis, erosions, or neutrophil influx into the joints, as compared to CIA controls. This is in complete contrast to animals infected with the live wild-type strain (W83), suggesting that PPAD likely contributes significantly to the pathogenesis of the disease.

The enzyme by itself is immunogenic in mice, as antibodies against PPAD were detected in animals inoculated with the wild-type strain, W83 [35••]. Moreover, as mentioned before, the anti-PPAD antibody response is unique to RA patients, suggesting that PPAD may constitute a neo-antigen in RA [32••]. Nevertheless, it is unlikely that this response contributes to the aggravation of CIA by *P. gingivalis* because the level of anti-PPAD antibodies was very low. By contrast, the level of antibodies to citrullinated peptides (aCCP) and to enolase-derived citrullinated epitope (CEP-1), both of which are implicated in autoimmunity in RA, strongly increased in CIA mice infected with wild-type *P. gingivalis* [35••]. Finally, demonstrating the role of autoantibody production in the pathophysiology of CIA, *P. gingivalis* infection increased the serum levels of specific IgG against CII, in contrast to either ΔPPAD or CIA control mice.

**Fig. 1:**
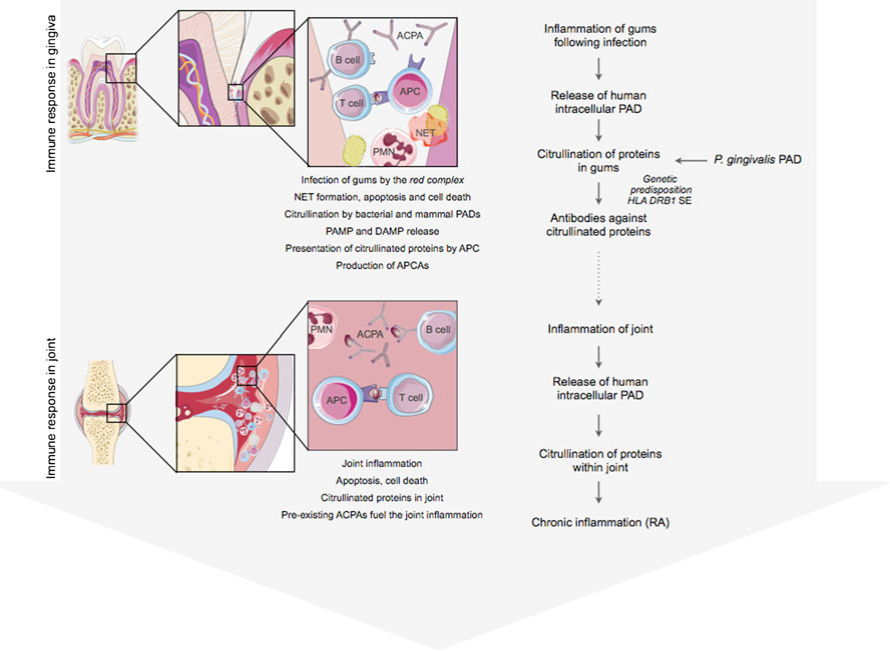
A model of periodontitis-initiated pathogenesis of rheumatoid arthritis (*RA*) initiated by PPAD/PAD-catalysed modification of proteins in the inflamed periodontal tissue and driven by autoimmunity against citrullinated epitopes joints. Figure was created using Servier Medical Art

Collectively, aggravation of these RA-associated pathologies is dependent on PPAD activity, which, either directly or indirectly (via the enhancement of inflammatory reactions and the release of host PADs), leads to the generation of citrullinated neo-epitopes, thus stimulating autoantibody production. In this context, one may hypothesise that pathological citrullination of proteins in gingival tissues by PPAD, within a chronic inflammatory environment in which the immune system is stimulated by bacteria-derived danger signals such as LPS, fimbriae, and peptidoglycan, breaks immune tolerance to citrullinated proteins and peptides, priming autoimmunity in a subset of patients with RA [36] (Fig. 1).

Notably, human PADs, PPAD, and citrullinated proteins have been detected in the oral mucosa and periodontium [33]. Thus, in periodontitis, the orchestrated citrullination of host and/or bacterial proteins in vivo could trigger a loss of tolerance to structurally similar host proteins, resulting in the expression of ACPA.

## Conclusions

Together, PD and RA are responsible for significant loss of function and morbidity in a large percentage of the population worldwide. Therefore, for many years, scientists have tried in vain to find a mechanistic link between both diseases in the hope of facilitating the development of a novel, effective treatment for both diseases. Based on these latest discoveries, we believe that PPAD could be a target for successful therapy or prevention of these crippling diseases.
